# Efficient RNA interference in patients' acute lymphoblastic leukemia cells amplified as xenografts in mice

**DOI:** 10.1186/1478-811X-10-8

**Published:** 2012-03-26

**Authors:** Ines Höfig, Harald Ehrhardt, Irmela Jeremias

**Affiliations:** 1 Department of Gene Vectors, Helmholtz Center Munich - German Research Center for Environmental Health, Marchioninistr. 25, 81377 Munich, Germany; 2 Division of Neonatology, University Children's Hospital, Ludwig-Maximilian-University, Marchioninistr.15, 81377 Munich, Germany; 3 Department of Oncology/Hematology, Dr. von HaunerschesKinderspital, Lindwurmstr. 4, 80337 Munich, Germany

## Abstract

**Background:**

Signaling studies in cell lines are hampered by non-physiological alterations obtained in vitro. Physiologic primary tumor cells from patients with leukemia require passaging through immune-compromised mice for amplification. The aim was to enable molecular work in patients' ALL cells by establishing siRNA transfection into cells amplified in mice.

**Results:**

We established delivering siRNA into these cells without affecting cell viability. Knockdown of single or multiple genes reduced constitutive or induced protein expression accompanied by marked signaling alterations.

**Conclusion:**

Our novel technique allows using patient-derived tumor cells instead of cell lines for signaling studies in leukemia.

## Introduction

Characterization of intracellular signaling mechanisms is crucial for the understanding of tumor development and for the design of novel strategies in anti-tumor therapy. For practical reasons, signaling studies are mainly performed in cell lines established from human tumors decades ago and are prone to non-representative mutations [1,2]. Example given, more than 50% of ALL cell lines inherit mutations of p53, while less than 5% of primary pediatric samples at initial diagnosis do [3-5].

To overcome these limitations, several groups had successfully transfected primary leukemia cells. Best results were obtained in samples from adult patients with chronic leukemias [6-8]. In contrast, in acute leukemias, rapid onset of therapy impedes repetitive sampling and reliable results of molecular experiments in primary cells. In pediatric leukemias, small sample volumes generally disable molecular work on these primary cells. For the transfection of childhood ALL samples by nucleofection, so far transgene expression was studied in detail rendering varying results between 1% and 62.3% [9].

Acute leukemia samples can be amplified in severely immuno-compromised mice. The xenograft mouse model of acute leukemia has been characterized to enable frequent engraftment with little genetic and phenotypic alterations upon passaging through mice [10]. The regular detection of CD surface markers revealed stable phenotypes over several passages [11].

Here, we describe a new method which will routinely allow repetitive and reliable molecular signaling studies on patient-derived childhood ALL cells amplified in NOD/SCID mice. The aim was to establish a suitable transfection technique to perform knockdown experiments in patient-derived acute lymphoblastic leukemia (ALL) cells, instead of ALL cell lines.

## Results

We used xenografted cells from n = 11 children with different subtypes and risk status of ALL (Table 1). All samples were successfully re-passaged in NOD/SCID mice, a model with minor clonal selection in ALL [12]. Samples were amplified in mice and used as fresh isolates from different passages without feeder cell co-cultivation as frequently seen. Persistence of CD surface markers was insured by testing before and after passage in NOD/SCID mice as described in the Methods section (data not shown).

**Table 1:** Patient characteristics of leukemia samples xenografted in NOD/SCID mice

Sample	Isolated from	Type of leukemia	Age (years)	Gender	State of disease	Cytogenetic characteristics
ALL-50	PB	pre-B-ALL	7	f	ID	n.a.
ALL-53	BM	pre-B-ALL	14	f	R	n.a.
ALL-54	BM	pre-B-ALL	3	f	ID	not aberrant
ALL-177	BM	pre-B-ALL	8	f	ID	TEL/AML1; del12p
ALL-199	BM	pre-B-ALL	8	f	R	trisomy 21
ALL-10S	BM	pre-B-ALL	4	f	ID	MLL-AF9 rearrangement
ALL-188	BM	pre-B-ALL	9	f	ID	TEL/AML1
ALL-4S	PB	T-ALL	4	m	ID	t(11;14);(p32;q11)
ALL-202	PB	T-ALL	9	m	ID	del9p
ALL-168	BM	pre-B-ALL	5	f	ID	der(19)t(1;19)(q23;p13)
ALL-169	BM	pre-B-ALL	18	f	ID	n.a.

*PB *peripheral blood, *BM *bone marrow, *(pre-)B-ALL *(precursor) B-cell acute lymphoblastic leukemia, *T-ALL *T-cell acute lymphoblastic leukemia, *f *female, *m *male, *ID *initial diagnosis, *R *relapse, *n.a*. no data available

### Optimization of the transfection of patient-derived childhood ALL cells

**Figure 1:**
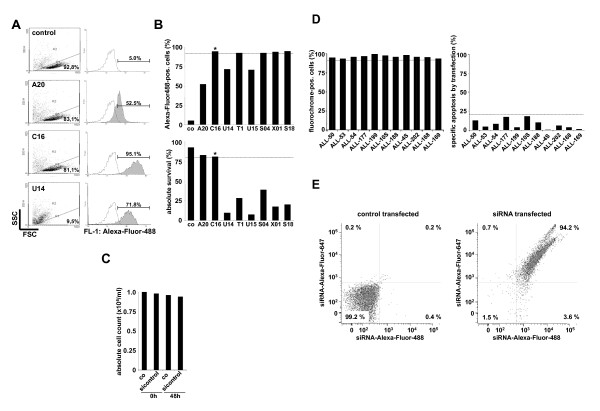
**Optimization of the transfection efficiency of patient-derived childhood ALL cells**. A) ALL cells of patient ALL-50 were xenografted and amplified in NOD/SCID mice, isolated from spleens and transfected by nucleofection with a control siRNA oligonucleotide conjugated to Alexa-Fluor-488 using different programs. After 48 h of in vitro culture, apoptosis was measured by forward side scatter analysis and transfection efficiency in living cells by the shift of fluorescence intensity in channel 1 in FACscan. Presented are original results of 4 different transfection conditions for sample ALL-50. B) The data of eight different transfection conditions (X-Axis indicates the different programs offered by the company) are summarized for transfection efficiency and impact of transfection on survival of ALL cells for sample ALL-50. * highlights the program used for further experiments. C) Absolute cell count was determined in ALL-50 cells untreated (co) and transfected with the dye-conjugated control sequence (sicontrol) at 0 and 48 h. D) Transfection efficiency and cell viability was determined in overall n = 11 different xenograft samples using program C16 as in Figure 1B. E) ALL cells from patient ALL-50 were simultaneously transfected with a control siRNA against Lamin conjugated to Alexa-Fluor-488 and siRNA control oligonucleotide conjugated to Alexa-Fluor647 as in Figure 1B using program C16.

Within the many transfection techniques available, electroporation using nucleofection rendered the most outstanding results in patient-derived ALL cells (data not shown). To optimize the transfection, 5 million cells were used within different conditions and protocols currently used for leukemic cell lines or primary lymphocytes. Best results were performed using nucleofection protocols for primary B- and T-cells. The programs differ in intensity (increasing from A to X) and the duration of the electric impulse (increasing from 1 to 25). Constantly high transfection efficiencies above 90% combined with low impact on spontaneous apoptosis (< 20% additional apoptosis by transfection) were obtained using a long pulse of low intensity (program C16) and the buffers for transfection of normal B- and T-cells (Figure 1A and 1B). Beyond viability, the absolute number of viable cells remained unchanged (Figure 1C). The optimized setting was successfully applied in all n = 11 xenografted patient samples with only marginal variations in transfection efficiency and cell viability (Figure 1D). Simultaneous transfection of two different siRNA sequences conjugated to non-overlapping dyes rendered transfection efficiencies similar to the single transfection (Figure 1E and data not shown).

The siRNA delivery by nucleofection leads to stable and reproducible results for a cohort of xenografted ALL samples.

### Efficient knockdown of target genes in patient-derived ALL cells

**Figure 2:**
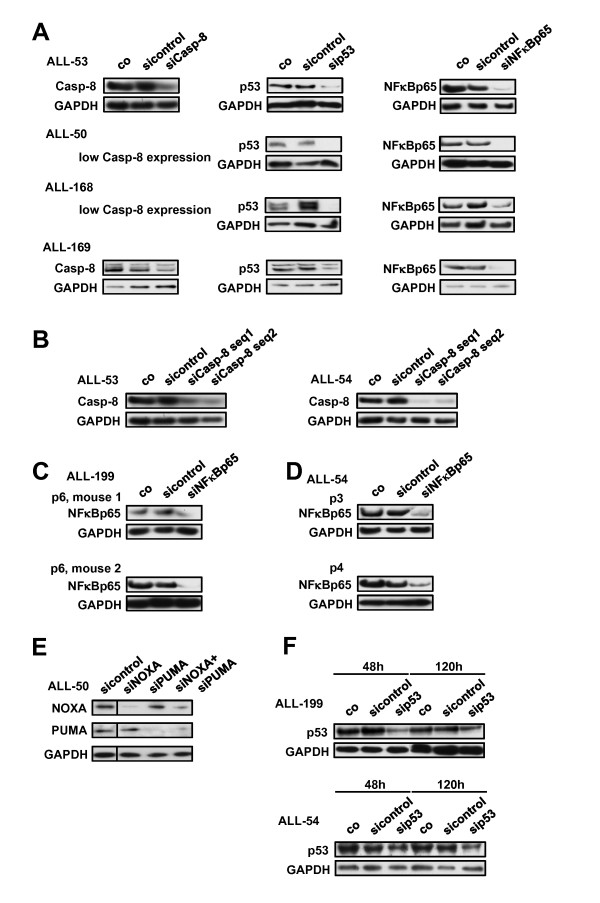
**Efficient knockdown of target genes in patient-derived childhood ALL-cells**. A) ALL cells from sample ALL-53, ALL-50, ALL-168 and ALL-169 were transfected with siRNA against Caspase 8, p53 or NFκBp65 as in Figure 1C. Cells were harvested 48 h after transfection and Western Blot analysis of total cellular protein was performed. GAPDH served as loading control. Where indicated, xenograft samples did express only low levels of Caspase-8. B) ALL cells from sample ALL-53 (left panel) and ALL-54 (right panel) were transfected as in Figure 2A using two different siRNA sequences targeting Caspase-8. C) ALL cells of sample ALL-199 were repetitively xenografted within the identical passage (p6) and treated and analyzed as in Figure 2A. D) ALL cells of sample ALL-54 were xenografted in passage 3 (upper panel) and 4 (lower panel) and treated and analyzed as in Figure 2A. E). ALL cells from sample ALL-50 were simultaneously transfected and analyzed as in Figure 2A using siRNAs against NOXA and PUMA. F). ALL-199 (upper panel) and ALL-54 (lower panel) were transfected with siRNA against p53 as in Figure 2A and analyzed at 48 and 120 h for the stability of the knockdown.

We used siRNAs to knock down p53 and its target genes Caspase-8, NOXA, PUMA and NFkBp65. Knockdown efficiency was confirmed on protein level in all samples and all five siRNAs tested rendered reproducible results (Figure 2A, Additional file 1: Figure S1A and data no shown). All siRNA sequences had been validated in JURKAT leukemia cell lines before. siRNAs were cloned into the pRetro or pSuperior vectors as shRNAs and were introduced into JURKAT cells by nucleofection followed by enrichment of transfected cells using Puromycin. When ever stable expression of the corresponding shRNA induced a > 90% knockdown of the target protein, the siRNA induced efficient knockdown in primary or xenograft cells. The knockdown efficiency was further verified for different siRNA sequences targeting the identical RNA (Figure 2B). Stability and reproducibility of the efficiency of the described technique was verified by repetitive analyses of xenografted cells from different mice within the identical passage (Figure 2C) and for different passages (Figure 2D).

Beyond single targets, successful double knockdown was obtained by targeting two proteins and yielded a simultaneously diminished expression of both proteins (Figure 2E and Additional file 1: Figure S1B). In selected samples with high viability over prolonged periods of time in cell culture (> 70% at 120 h), stability of knockdown was detected for up to 120 h (Figure 2F and data not shown).

### Knockdown of target genes altered signaling in patient-derived ALL cells

In a next step, the functional impact of the efficient siRNA delivery for the samples from Figure 2 and Additional file 1: Figure S1 was tested for the intracellular apoptosis signaling transduction.

**Figure 3:**
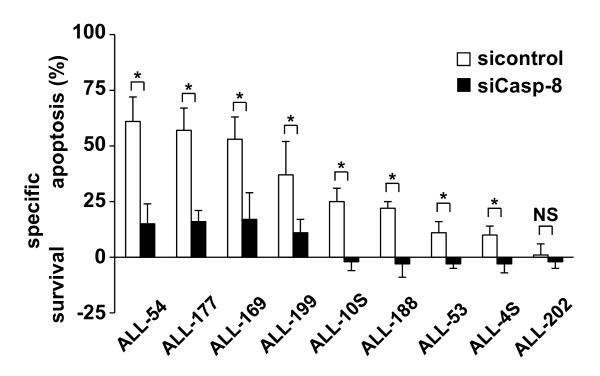
**Functional impact of the efficient siRNA interference**. n = 9 ALL cells expressing Caspase-8 from Figure 2A and Additional file 1: Figure S1A were transfected with siRNA against Caspase 8 as in Figure 2A and Additional file 1: Figure S1A and stimulated with TRAIL (100 ng/ml) 48 h after transfection. Cell death induction was determined after another 48 h. Depicted are mean values and SD of at least three independent experiments performed in duplicates. *p < 0.05, paired *t*-test, NS = not significant.

Caspase-8 has been repetitively shown to be essential for efficient apoptosis induction by the death inducing ligand TRAIL [13-15]. The functional impact of the efficient downregulation of Caspase-8 by siRNA interference as depicted in Figure 2A and Additional file 1: Figure S1A was verified after stimulation with TRAIL in n = 9 different xenografted samples in at least 3 independent experiments which expressed detectable levels of Caspase-8 (Figure 3). As we had published before, the efficient knockdown of Caspase-8 and the following stimulation with TRAIL was associated with increased survival in culture compared to the control cells in some of the samples investigated [14,15].

### Knockdown of target genes alters protein regulation and function in patient-derived ALL cells

So far, the efficiency of our technique was evaluated for basal protein expression. In a next step, we evaluated the effect of siRNA-mediated knockdown on protein regulation in patient-derived ALL cells.

**Figure 4:**
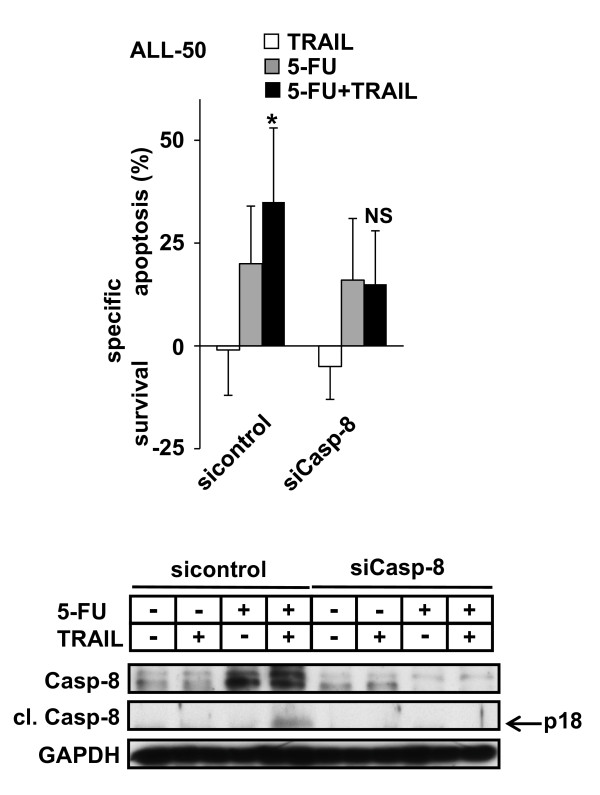
**Efficient inhibition of protein regulation**. ALL cells of patient ALL-50 were transfected as in Figure 1C with a non-specific sequence (sicontrol) or siRNA against Caspase-8 (Casp-8) followed by stimulation with 5-Fluorouracil (5-FU; 30 μM) and TRAIL (1 μg/ml)) 6 h later for another 72 h. In parallel, Western Blot analysis was performed of cells incubated with 5-FU for 48 h. GAPDH served as loading control. One representative blot is shown. Depicted are mean values and SD of 4 independent experiments performed in duplicates. *p < 0.05, paired *t*-test, combined stimulation compared to the additive result of both single agents, NS = not significant.

ALL-168 and ALL-50 showed minor constitutive expression of Caspase-8 and the expression level was augmented by the addition of the cytotoxic drug 5-Fluorouracil according to recent cell line data [13]. On a functional level, 5-Fluorouracil induced slight upregulation of Caspase-8 and sensitized cells towards TRAIL-induced apoptosis when TRAIL was applied in high concentrations. Transfection of siRNA targeting Caspase-8 completely abrogated 5-Fluorouracil-induced upregulation of Caspase-8 in patient-derived ALL-50 and ALL-168 cells. In the absence of upregulation of Caspase-8 due to transfection of Caspase-8 targeting siRNA, Fluorouracil was no longer capable to sensitize towards TRAIL-induced apoptosis (Figure 4 and Additional file 1: Figure S2).

On a broader level, these data prove that efficient siRNA delivery in patient-derived childhood ALL cells induces significant functional alterations which can be achieved either upon knockdown of constitutively expressed genes or by inhibition of protein regulation. Taken together, we have successfully established a method allowing reliable RNAi-based signaling studies in patient-derived pediatric ALL cells.

### Applicability of the described technique to primary ALL cells

**Figure 5:**
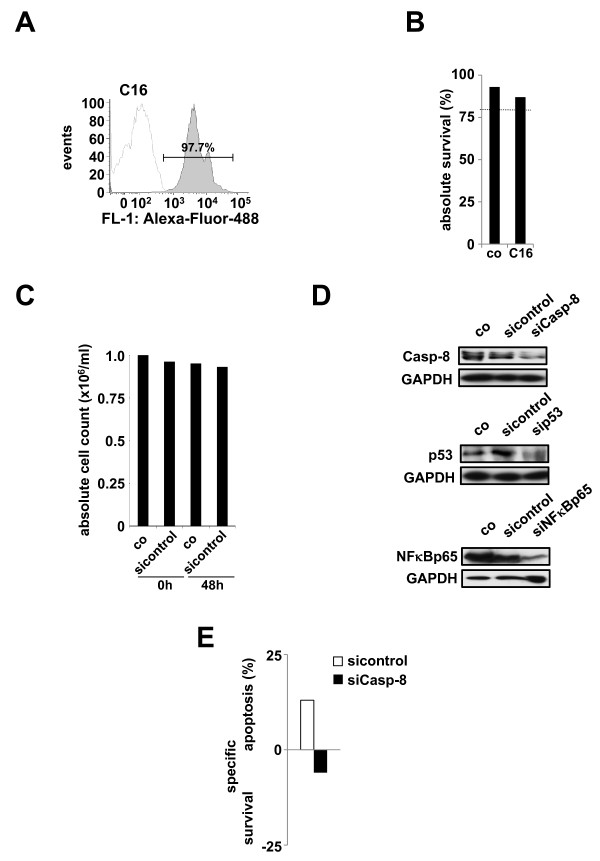
**Applicability of the described technique to primary patient-derived childhood ALL-cells**. A-C)Freshly isolated ALL cells from a 8 year old girl at initial diagnosis of T-ALL were transfected and analyzed as in Figure 1A-C. D) Primary ALL cells from Figure 5A were transfected with specific siRNAs as in Figure 2A. E) Primary ALL cells from Figure 5A were transfected and stimulated with TRAIL as in Figure 3.

So far primary childhood ALL cells were only used after xenograft transplantation due to the usually limited number of cells available. As proof of principle, we performed the identical experimental procedure in primary childhood T-ALL cells isolated from peripheral blood of a 8 year old girl at initial diagnosis. Using a dye-conjugated control siRNAnucleofected with program C16, we were able to demonstrate a comparable transfection efficiency, high viability of the cells and unchanged absolute cell count comparable to the xenograft experiments (Figure 5A-C). Similarly, siRNA transfection using siRNAs against Caspase-8, p53 and NFκB rendered efficient knock-down of target proteins (Figure 5D) accompanied by marked functional alterations studied using Caspase-8 and TRAIL (Figure 5E).

The data obtained in this primary ALL sample suggest that the described technique is well applicable not only to xenografted primary ALL cells but although to at least some primary ALL samples.

## Conclusions

We present a novel method allowing repetitive molecular signaling studies in pediatric ALL cells from individual children. The focus of our method are studies of signaling pathways which are not adequately modeled in ALL cell lines and require patient-derived tumor cells, such as the p53 network. The combination of amplification of these cells in immune-incompetent mice and the presented transfection technique allows molecular signaling studies (i) in patient-derived pediatric samples, where small volumes are notoriously restrictive; (ii) in cells which resemble primary tumor cells substantially better than established cell lines do [11,12]; (iii) repetitively on highly viable cells allowing reliable results. To return to the discussed difficulty of p53 signaling studies in leukemia cell lines, the presented technique will enable routine investigation of primary childhood ALL cells that have been performed so far mainly in childhood ALL cell lines with restricted relevance to the clinical setting due to inherited mutations i.e. in CEM and JURKAT leukemia cells [13,16].

Upon isolation from spleen or bone marrow of mice, patient-derived cells can be used freshly for repetitive experiments and show better viability compared to primary samples due to more favorable handling and shipping. To our experience, cell viability is sufficient for transfection experiments in the majority of patient-derived samples freshly isolated from mice. The data obtained in the primary childhood ALL sample suggest that the described technique is as well applicable to freshly isolated primary ALL samples. It remains to be tested, whether our method generally facilitates transfection of freshly isolated, primary ALL cells, e.g. from adult patients and other types of primary leukemia cells which were not studied so far using this technique.

## Methods

### Materials

TRAIL was obtained from PeproTech (Hamburg, Germany), all further reagents were obtained from Sigma-Aldrich (St. Louis, MO). For Western Blot analysis, the following antibodies were used: anti NFκBp65 and anti p53 from Santa Cruz Biotechnology Inc. (Santa Cruz, CA); anti PUMA from Cell Signaling Technology Inc. (Danvers, MA); anti GAPDH from Thermo Fisher (Waltham, MA); anti NOXA from Calbiochem (San Diego, CA); anti Caspase-8 from Alexis Corp. (Lausen, Switzerland).

For flow cytometric analysis of CD surface marker stability, the following antibodies were used: anti CD4-PE from DAKO (Hamburg, Germany), anti CD5-PeCy5.5, anti CD7-APC, anti CD10-APC, anti CD19-PeCy5.5, anti CD22-PE and anti HLA-DR-PeCy5.5 from Life Technologies (Darmstadt, Germany), anti CD19-FITC, anti CD20-FITC, anti CD34-PE and anti CD38-PE from BD Biosciences (San Jose, CA).

### Amplification of primary childhood ALL cells in NOD/SCID mice

Informed consent was obtained from all patients and experiments were performed according to the declaration of Helsinki as approved in written form by the ethical committee of the medical faculty of the Ludwig Maximilians University Munich (LMU 068-08) and the Children's Hospital of the TU Munich (TU 2115/08). Primary ALL blasts were obtained from children treated at the Ludwig Maximilians University Children's Hospital or the children's hospital of the TU Munich. Tumor cells were isolated from blood or bone marrow samples.

Animal work was approved by the Regierung von Oberbayern (55.2-1-54-2531-2-07). The xenograft NOD/SCID mouse model was performed as described by others [17-19]. Fresh primary childhood ALL cells were isolated by Ficoll gradient centrifugation for 30 min at 500 g from peripheral blood or bone marrow aspirates that had been obtained from leftovers of clinical routine sampling followed by two washing steps in PBS before resuspension in cell culture medium. 10 million ALL cells were injected into 6-8 weeks old NOD/SCID mice via the tail vain. Engraftment was monitored by flow cytometry and measurement of human cells in peripheral blood. Engrafted human ALL cells were isolated from spleens of diseased mice by pressing through a cell strainer (BD Biosciences) and Ficoll gradient centrifugation. Cells were separated and simultaneously injected into next generation of mice and subjected to in vitro experiments. Regular detection of CD surface markers revealed stable phenotypes of the samples over all passages [11].

### Transfection and stimulation of patient-derived ALL cells

For nucleofection (AmaxaNucleofector, Lonza, Basel, Switzerland), 5 million cells were used per reaction. Patient-derived ALL cells were resuspended in 100 μl pre-warmed buffer from the nucleofector kit for human B/T-cells plus 5 μlsiRNA oligonucleotides (20 μM). The following siRNAs were used: silencer validated siRNA against p53 (5'-GGGUUAGUUUACAAUCAGC-3', Ambion, Austin, TX), siRNA against NOXA (5'-GUCGAGUGUGCUACUCAACU-3'); siRNA against PUMA (5'-UCUCAUCAUGGGACUCCUG-3'; siRNA against Caspase-8 (5'-GCUCUUCC GAAUUAAUAGATT-3', second siRNA against Caspase 8 (siCasp8_2; 5'-GCUCUUCCGAAUUAAUAGATT-3') and siRNA against NFκBp65 (all from MWG Biotech, Ebersberg, Germany) and All Star negative control siRNA conjugated with Alexa-Fluor-488 or Alexa-Fluor-647 (Qiagen, Hilden, Germany). For double transfections, siRNA against lamin conjugated to Alexa-Fluor-488 (analyzed in FITC channel) and negative control siRNA conjugated with Alexa-Fluor-647 (APC-channel) was analyzed using a LSR II flow cytometer (BD Biosciences) and FlowJo (TreeStar, Ashland, OR) software version 8.3. After transfection, cells were incubated 5 min at room temperature in the nucleofection cuvette, then transferred to 5 ml pre-warmed RPMI medium supplemented with 20% FCS, 1% penicillin/streptomycin, 1% gentamycin, 6 μl/ml mixture of insulin, transferrin and selenium (Invitrogen, Carlsbad, CA), 1 mM sodium pyruvate and 50 μM 1-thioglycerole (Sigma-Aldrich, St. Louis, MO). After transfection with fluorochrome-conjugated negative control siRNA, cells were washed twice in PBS prior to flow cytometric determination of transfection efficiency. Stimulation experiments were started 6 or 48 h after transfection.

### Western blot and measurement of apoptosis

Western Blot analysis was performed out of total cellular lysates using the following total cell lysis buffer: 20 mMTris-HCl (pH 7.5), 150 mMNaCl, 1 mM Na_2_EDTA, 1 mM EGTA, 1% Triton, 2.5 mM sodium pyrophosphate, 1 mM beta-glycerophosphate, 1 mM Na_3_VO_4 _supplemented with 10 μg/ml protease inhibitor cocktail set I (Calbiochem). Lysates were separated by SDS-page gel electrophoresis followed by transfer onto a nitrocellulose membrane and detection of the primary antibody by a HRP-conjugated secondary antibody. Apoptosis was measured by forward side scatter analysis and precision of this technique confirmed by Annexin V and propidium iodide double staining according to the manufacturers instructions using FACscan or LSR II flow cytometry and Cell Quest Pro (BD Biosciences) software version 3.2.1.

### Statistical analysis

Specific apoptosis was calculated as [(apoptosis of stimulated cells at end point minus apoptosis of unstimulated cells at end point) divided by (100 minus apoptosis of unstimulated cells at end point) times 100]. Whenever indicated, paired *t*-test was performed to detect statistically significant differences out of at least three independent experiments with two technical replicates. Statistical significance was accepted with p-values < 0.05.

## Competing interests

The authors declare that they have no competing interests.

## Authors' contributions

IH and HE performed the experiments and prepared the figures and data analyses. HE and IJ designed the research, provided administrative support, and wrote the paper. All authors read and approved the final manuscript.

## Supplementary Material

Additional file 1 **Figure S1**. Efficient knockdown of target genes in patient-derived childhood ALL-cells ALL cells from n = 7 further patients were transfected as in Figure 2A. ALL cells from sample ALL-169 were treated and analyzed as in Figure 2E. Figure S2. Efficient inhibition of protein regulation. ALL-168 cells were treated and analyzed as in Figure 4. Statistical analysis was performed out of n = 8 independent experiments. *p *< 0.05, paired t-test, NS = not significant. Click here for file
