# Supplementary material for: Efficient RNA interference in patients' acute lymphoblastic leukemia cells amplified as xenografts in mice
Source: Cell Commun Signal. 2012 Mar 26;10:8. doi: 10.1186/1478-811X-10-8 (PMC3349556; doi:10.1186/1478-811X-10-8)
Supplement: Additional file 1 — Figure S1. Efficient knockdown of target genes in patient-derived childhood ALL-cells ALL cells from n = 7 further patients were transfected as in Figure 2A. ALL cells from sample ALL-169 were treated and analyzed as in Figure 2E. Figure S2. Efficient inhibition of protein regulation. ALL-168 cells were treated and analyzed as in Figure 4. Statistical analysis was performed out of n = 8 independent experiments. p < 0.05, paired t-test, NS = not significant. [file 1478-811X-10-8-S1.PPT]

## Slide 1
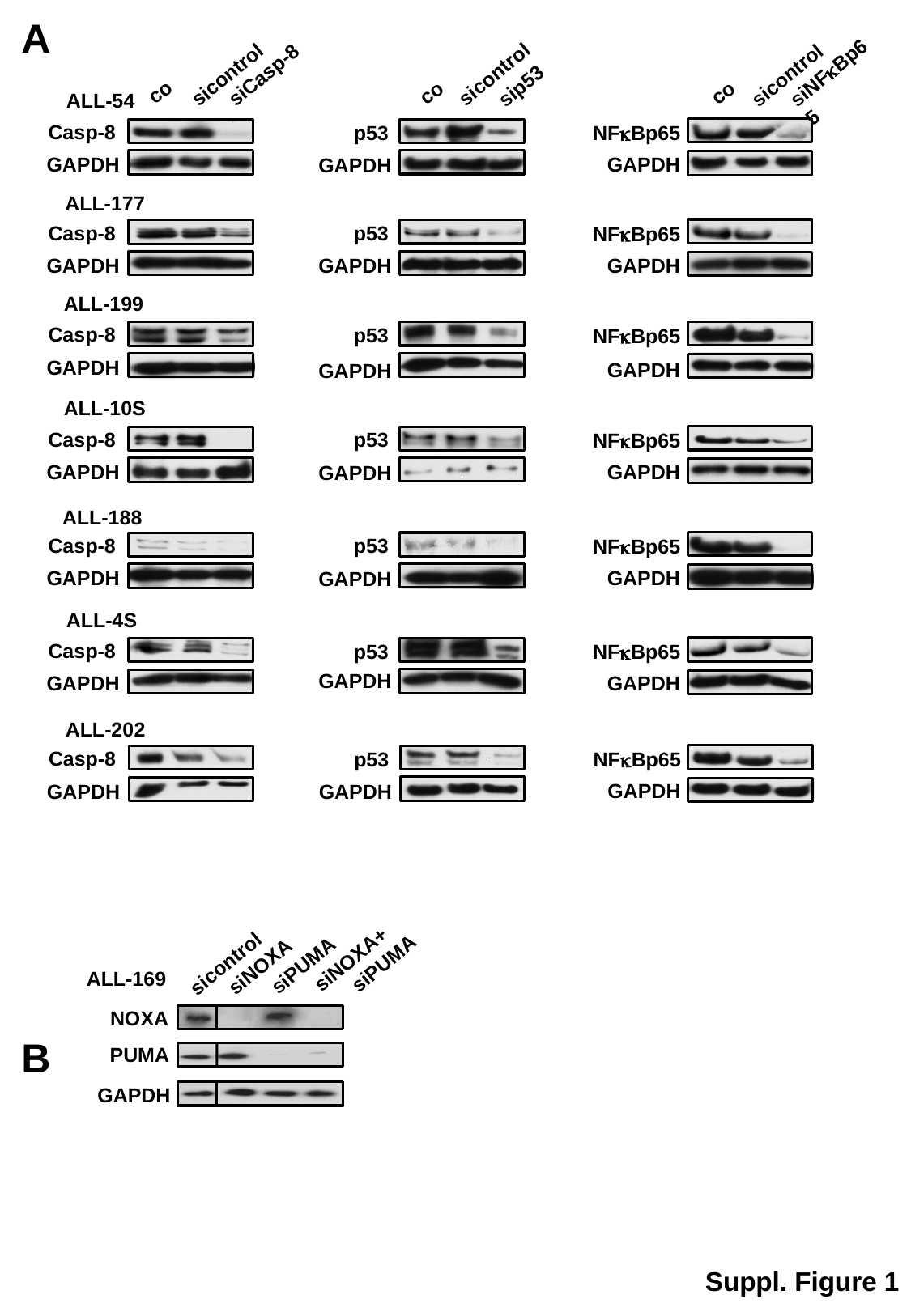

A
 B
siNFκBp65
siCasp-8
sicontrol
sip53
sicontrol
sicontrol
 co
 co
 co
ALL-54
Casp-8
p53
NFκBp65
GAPDH
GAPDH
GAPDH
ALL-177
Casp-8
p53
NFκBp65
GAPDH
GAPDH
GAPDH
ALL-199
Casp-8
p53
NFκBp65
GAPDH
GAPDH
GAPDH
ALL-10S
Casp-8
p53
NFκBp65
GAPDH
GAPDH
GAPDH
ALL-188
Casp-8
p53
NFκBp65
GAPDH
GAPDH
GAPDH
ALL-4S
Casp-8
p53
NFκBp65
GAPDH
GAPDH
GAPDH
ALL-202
Casp-8
p53
NFκBp65
GAPDH
GAPDH
GAPDH
siNOXA+
 siPUMA
siPUMA
siNOXA
sicontrol
ALL-169
NOXA
PUMA
GAPDH
Suppl. Figure 1

## Slide 2
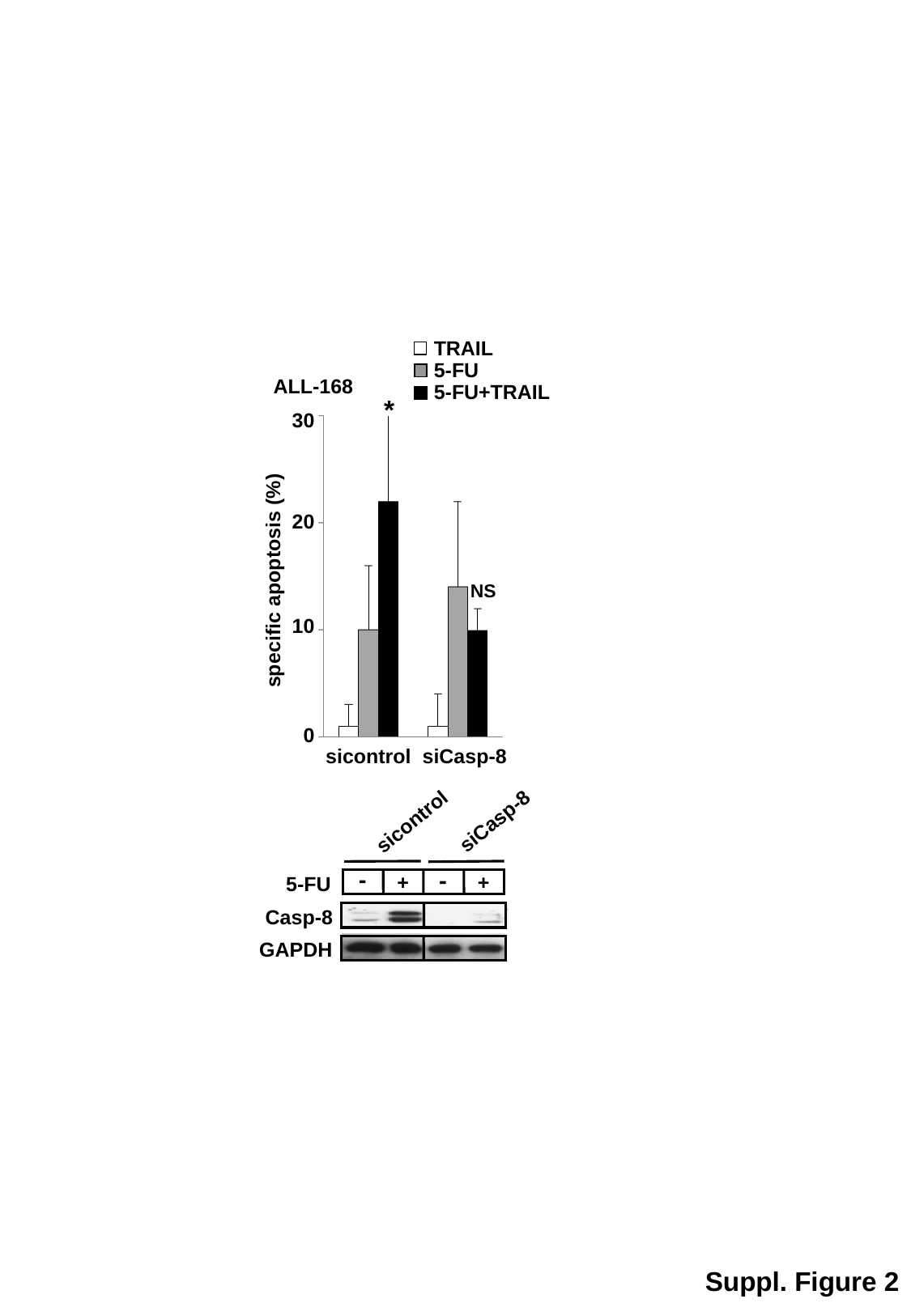

TRAIL
5-FU
5-FU+TRAIL
ALL-168
*
30
20
10
0
specific apoptosis (%)
NS
 sicontrol siCasp-8
siCasp-8
sicontrol
-
-
+
+
5-FU
Casp-8
GAPDH
Suppl. Figure 2
